# The effects of colour and temporal frequency of flickering light on variability of the accommodation response in emmetropes and myopes

**DOI:** 10.1186/s12886-021-01856-z

**Published:** 2021-02-17

**Authors:** Liyue Zhang, Dongyu Guo, Chen Xie, Yingying Wen, Xuhong Zhang, Le Jin, Jianping Tong, Ye Shen

**Affiliations:** Department of Ophthalmology, the First Affiliated Hospital, Zhejiang University School of Medicine, Hangzhou, People’s Republic of China

**Keywords:** Myopia, Accommodation, Light pollution, Flicker frequency, Flickering light

## Abstract

**Background:**

Myopia is hypothesized to be influenced by environmental light conditions. For example, it has been shown that colour and temporal frequency of flickering light affect emmetropisation in animals. Considering the omnipresence of flickering light in our daily life, we decided to analyze the effect of colour flickers on variability of the accommodation response (VAR) in emmetropes and myopes.

**Methods:**

We measured the dynamic accommodative responses of 19 emmetropic and 22 myopic adults using a Grand Seiko WAM-5500 open-field autorefractor. The subjects focused for more than 20 s on a black Snellen E target against three different backgrounds made up of three colour flicker combinations (red/green, red/blue and blue/green) and under five frequency conditions (0.20 Hz, 0.50 Hz, 1.00 Hz, 1.67 Hz, and 5.00 Hz).

**Results:**

Flicker frequency and colour both had a significant effect on VAR. Lower frequencies were associated with larger variability. Colour had an effect only at low frequencies, and red/blue colour flicker resulted in the largest variability. The variability in myopes were larger than those in emmetropes.

**Conclusions:**

These findings support the hypothesis that further studies on the colour and temporal frequency of flickering light can lead to a better understanding of the development and progression of myopia.

## Background

Myopia is an important global health problem that, by the year 2050, is projected to affect 49.8% of the world’s population [1–3]. Thus, it has gained the attention of many researchers who investigate its underlying causes [4, 5]. Recent studies have indicated that the spectral composition of environmental light provides one of the most important cues for the refractive development of the eye and light becomes a pollution to eyes [6, 7]. Today, with the development of modern artificial light sources, our eyes are being increasingly exposed to complex light stimuli. For example, coloured flickering light, which is quite different from natural light, is present in everyday life nowadays. Pictures change quickly in videos on the colourful screen and colourful lights shifted quickly in some places like concerts or bars are some kinds of flickering light. Animal studies have demonstrated a significant effect of colour flickers on refractive development, indicating that the eyes sense colour and temporal frequency of flickering light and use them as developmental cues [8–10].

However, the effect of colour flickers on human accommodation has not yet been explored in much detail. Longitudinal chromatic aberration (LCA) under composite coloured light conditions leads to long-wavelength light being focused behind the short-wavelength light on the retina, thus inducing a larger accommodative response. It has been shown that there is an accommodative difference of 1.26 D between wavelengths of 440 nm and 650 nm in humans [11]. The accommodative response shifts with the wavelength of the light. When the colour of the target was shifted between red and blue at low frequency (0.25 Hz), the accommodative response changed in different magnitudes, from about − 2.39 D to − 1.27 D, depending on the colour [12]. Thus, colour flickers can lead to a change in the accommodative stimulus, affecting the accommodative response, and resulting in accommodative fluctuations. However, when focusing on a stationary target, the accommodation of eyes is not stable but fluctuates over a small range of about ±0.5 D which we now call accommodative microfluctuations (AMFs) [13, 14]. Colour flickers may lead to a bigger accommodative fluctuation based on the inherent AMFs and enlarge the variability of the accommodation response (VAR). Recently, the association between AMFs and refraction was investigated by several studies [15–17]. Many researchers have found that AMFs in myopes are significantly larger than those in emmetropes and it has been speculated that AMFs may be a risk factor for the development and progression of myopia [15, 18–20]. We figure whether the VAR under colour flickers will also be different between myopes and emmetropes as AMFs are the base of accommodative fluctuation.

Colour flickers in our daily life may play a role in myopia development and progression, which has been implicated by animal studies [8–10]. However, very few studies so far focused on the effect of colour flickers on human accommodation, especially in both myopes and emmetropes. Therefore, in this study, we aimed to explore the relationship between colour and temporal frequencies of flickering light as well as the refractive error with the VAR in humans. We assessed accommodation response in emmetropes and myopes and focused on combinations of three colour conditions (red/blue, red/green and blue/green) at five temporal frequencies to investigate how colour and temporal frequency affect VAR in emmetropes and myopes.

## Methods

### Participants

Forty-one subjects aged 22–30 years participated in the study. All subjects had normal colour vision, less than 0.75 D of astigmatism, 0.0 logMAR visual acuity or better and no other ocular or systemic disease. Before the experiment, all subjects had been tested for subjective refraction and had undergone slit-lamp examination, fundus examination, chromoptometry, amplitude of accommodation examination, accommodative facility examination and visual fatigue questionnaire to exclude accommodative dysfunctions or any other disorders that could affect the study results.

The subjects were divided into two groups: an emmetropes (EMM) group (*n* = 19) and a myopes (MYO) group (*n* = 22). The EMM group comprised those subjects with a spherical equivalent refractive error (SE) between − 0.5 and + 0.5 D, while the MYO group had subjects with an SE between − 1.0 D and − 6.0 D. SE difference between two eyes of each subject was less than 1.0 D in both groups. The right eyes of all subjects were used in the analysis, and so all data pertain only to the right eye (Table 1).

**Table 1:** Details of the subject groups

Refractive group	EMM group	MYO group
Number of subjects	19	22
Age (years)	25.89 ± 1.15	25.55 ± 1.57
SE (D)	−0.11 ± 0.43	−4.13 ± 1.12 *

Data were analyzed with t-test. The asterisk indicates a significant difference between EMM group and MYO group in SE (*p* < 0.05).

The study was approved by the ethics committee of the First Affiliated Hospital, Zhejiang University School of Medicine, and was in accordance with the tenets of the Declaration of Helsinki. All subjects provided written consent. The study subjects were asked to stay away from alcohol and caffeine for more than 24 h before the study and to sleep for more than 7 h before the day of the study.

### Procedures

**Fig. 1:**
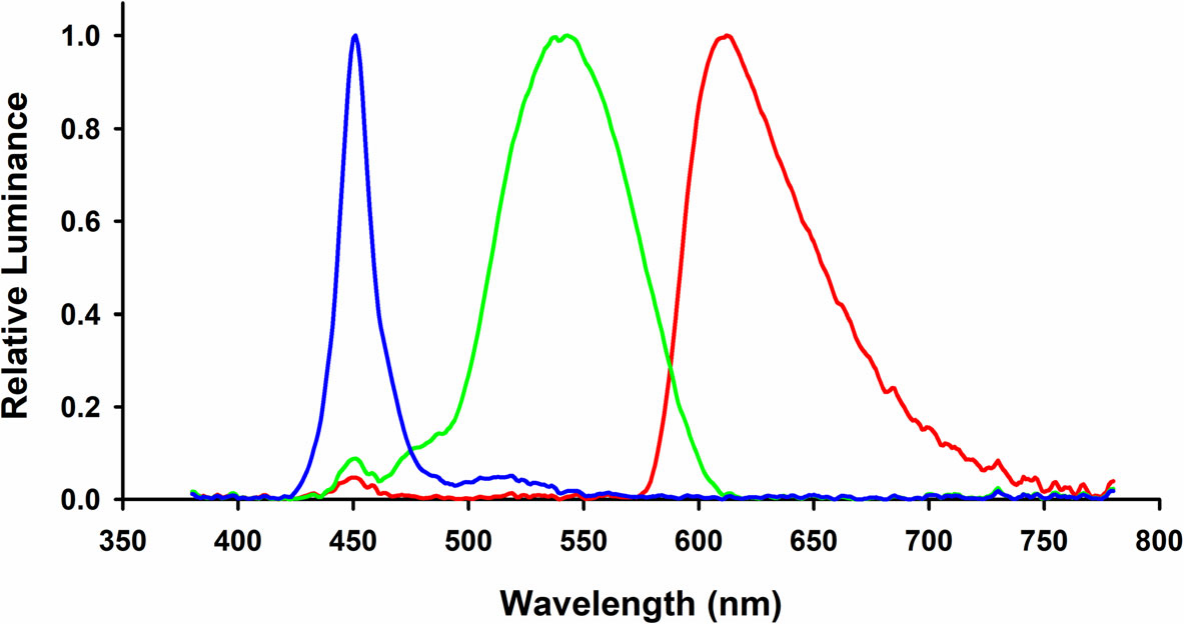
The spectra of the three background colours on the screen

The black Snellen E target of 0.1 logMAR size was displayed at the centre of a 12.3-in. laptop screen (Microsoft Surface Pro 7) with a resolution of 2736 × 1824 pixels at a distance of 33 cm, corresponding to an accommodative demand of 3 D. The background colours were changed between red (RGB: (250, 0, 0) with the wavelength of 611 nm), green (RGB: (0, 250, 0) with the wavelength of 547 nm), and blue (RGB: (0, 0, 250) with the wavelength of 463 nm) (Fig. 1). The room was dark, and so the screen was the only visible stimulus. Three types of colour flicker conditions were assessed in this study, namely, red/green (R/G), red/blue (R/B) and blue/green (B/G) flickers. The flicker frequencies used in the study were 0.20 Hz, 0.50 Hz, 1.00 Hz, 1.67 Hz and 5.00 Hz. The highest frequency was 5.00 Hz, which was the detection frequency limit of the autorefractor. The lowest frequency was adapted based on the findings of a previous study [12]. Three stable targets (none frequency) were also been tested which were R/G (250, 250, 0), R/B (250, 0, 250) and G/B (0, 250, 250).

All subjects from the MYO group were fully vision-corrected with soft contact lenses since a previous study had demonstrated that soft contact lenses did not influence AMFs [21]. Subjects inserted the lenses at least 30 min before the start of measurements to ensure that there was enough time for adaptation.

Subjects viewed the screen (angular subtense: 44.74°; average luminance of the background: 11.03 lx) monocularly, using only their right eye. The left eye was occluded. A head rest kept the subject’s head still. The target was aligned with the eye to ensure on-axis measurement. Eighteen target conditions were presented randomly with different colour combinations and frequencies. Subjects were told to concentrate on the E target, ensure that it was always in clear focus and view it continuously for more than 20 s. To minimize adaptation effects, all subjects were requested to close their eyes for a span of 2 min between tests.

The dynamic accommodative responses and the pupil size were recorded using the Grand Seiko WAM-5500 open-field autorefractor (Grand Seiko, Fukuyama City, Hiroshima, Japan) in high-speed mode, which enables continuous measurement of data every 0.2 s and possesses a sensitivity for accommodative responses as low as 0.01 D [22]. The customized software of the Grand Seiko WAM-5500 automatically removed blinking artefacts. We also removed the data before and after any abnormal responses. Ultimately, the accommodative responses in each condition over a period of 20 s were examined. VAR was quantified in terms of the standard deviation of the continuous accommodative responses of 20 s duration.

### Data analysis

Repeated-measures analysis of variance was carried out to test the VAR with refractive groups (EMM versus MYO) as between-subject factor, and frequency and colour as within-subject factors. When the data did not meet the spherical test, the Greenhouse–Geisser method was used for correction. Effect size was estimated using partial η^2^. Least significance difference (LSD) test was conducted for multiple comparisons. The statistical significance level was set to 0.05, and statistical power was set to 0.80. All analyses were performed using SPSS (version 20; Armonk, NY, USA).

## Results

**Fig. 2:**
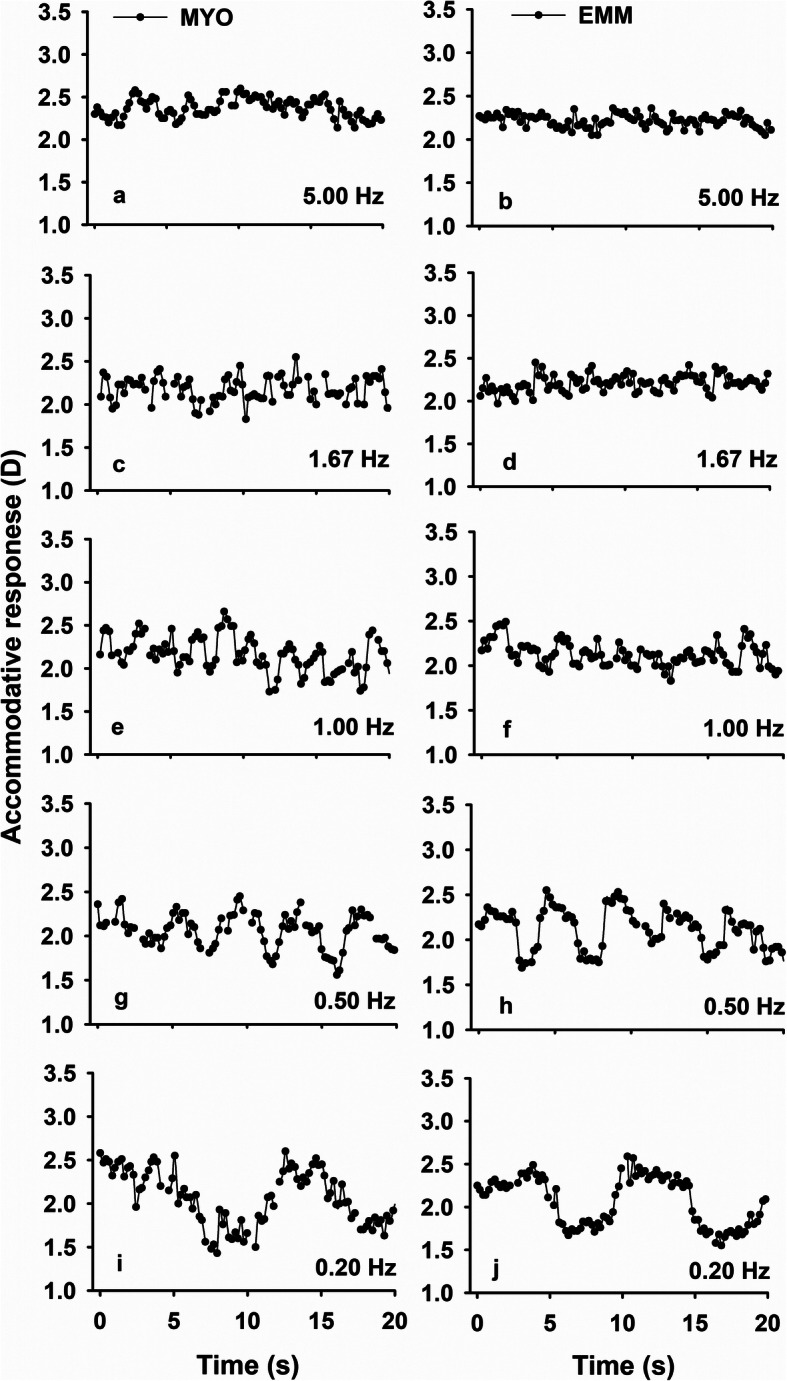
Typical accommodative responses of one subject from the myope (MYO) groups (**a**, **c**, **e**, **g**, **i**) and the emmetrope (EMM) groups (**b**, **d**, **f**, **h**, **j**) when viewing red/blue flicker targets with frequencies of 5.00 Hz, 1.67 Hz, 1.00 Hz, 0.50 Hz and 0.20 Hz, respectively

Figure 2 shows examples of the accommodative response of one subject from the EMM group and one from the MYO group for the R/B target condition. Subjects in both groups showed accommodative responses that fluctuated with the changes in background colour. However, for the same target condition, the variability of the accommodative responses in the MYO group was larger than in the EMM group.

**Fig. 3:**
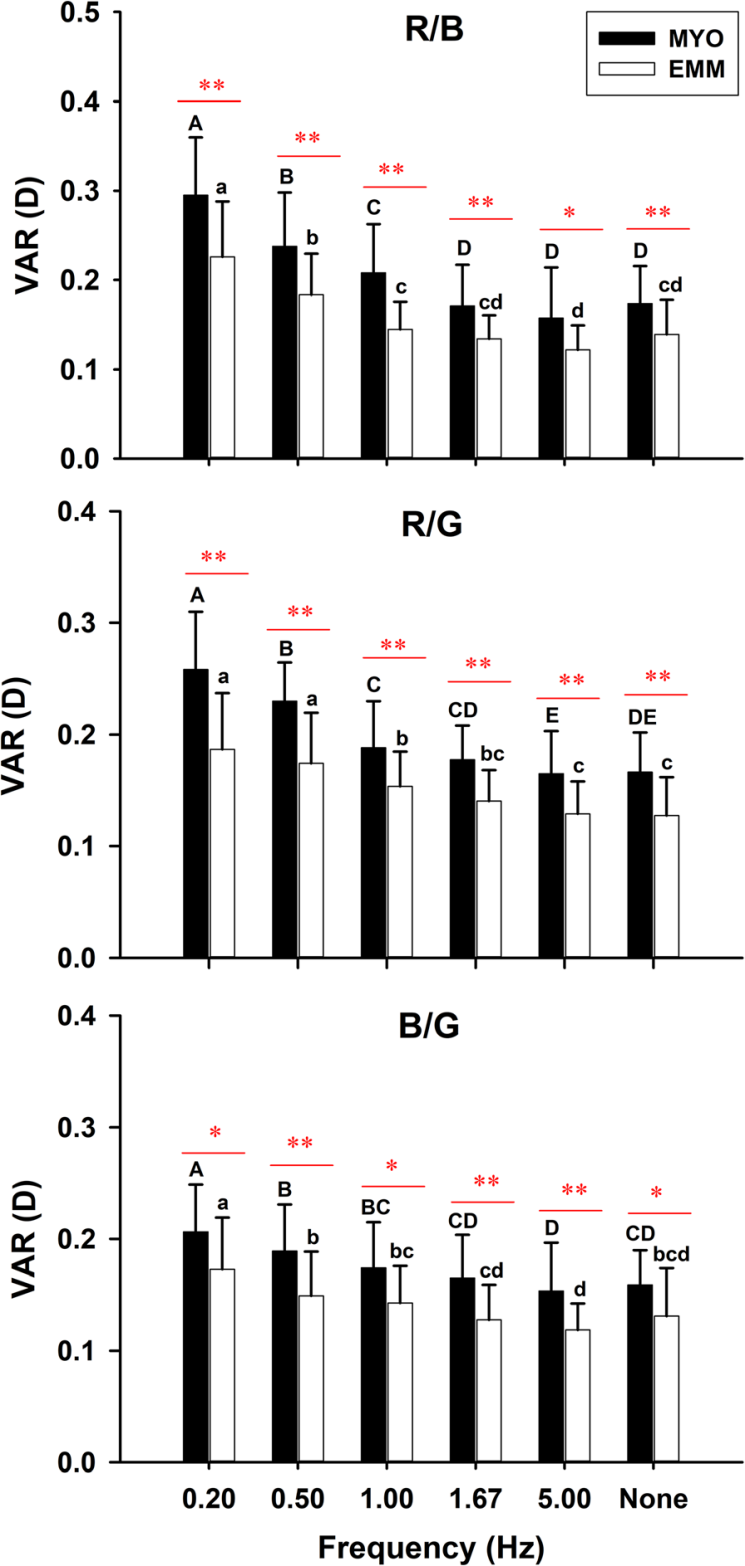
Effects of frequency of colour flickers on variability of accommodation response (VAR) in the emmetrope (EMM) and myope (MYO) groups under the different colour conditions. Different uppercase letters above the error bars indicate significant differences among frequencies in the MYO group. Different lowercase letters above the error bars indicate significant differences among frequencies in the EMM group. The above-line asterisks indicate significant differences between the EMM and MYO groups (LSD post hoc test; *p < 0.05, **p < 0.01). R/B indicates red/blue flicker, R/G indicates red/green flicker, and B/G indicates blue/green flicker

In general, VAR in the MYO group were significantly larger than those in the EMM group (*F*_1, 39_ = 23.488, *p* < 0.001, partial η^2^ = 0.376), and different frequencies and light colour both had significant effects on the VAR (frequency: *F*_3.15, 122.75_ = 95.739, *p* < 0.001, partial η^2^ = 0.424; colour *F*_2, 78_ = 28.680, *p* < 0.001, partial η^2^ = 0.711). VAR decreased gradually with increasing frequencies in both groups under all colour flicker conditions (Fig. 3; Table 2). A multiple comparison test also showed that VAR in the MYO group were significantly higher than those in the EMM group for all conditions (Fig. 3).

**Table 2:** Descriptive results of variability of accommodation response (VAR) in the emmetrope (EMM) and myope (MYO) groups under different colour conditions

Colour	Frequency (Hz)	Myopia (D)	Emmetropia (D)
R/B	0.20	0.295 ± 0.065	0.226 ± 0.062
	0.50	0.238 ± 0.060	0.183 ± 0.046
	1.00	0.208 ± 0.054	0.145 ± 0.031
	1.67	0.171 ± 0.046	0.134 ± 0.026
	5.00	0.158 ± 0.056	0.122 ± 0.027
	None	0.174 ± 0.042	0.139 ± 0.039
R/G	0.20	0.258 ± 0.052	0.188 ± 0.050
	0.50	0.230 ± 0.034	0.174 ± 0.045
	1.00	0.188 ± 0.042	0.153 ± 0.031
	1.67	0.177 ± 0.031	0.140 ± 0.028
	5.00	0.165 ± 0.038	0.129 ± 0.029
	None	0.166 ± 0.036	0.127 ± 0.034
B/G	0.20	0.206 ± 0.042	0.173 ± 0.046
	0.50	0.189 ± 0.042	0.149 ± 0.039
	1.00	0.174 ± 0.041	0.143 ± 0.033
	1.67	0.165 ± 0.038	0.128 ± 0.031
	5.00	0.153 ± 0.043	0.119 ± 0.024
	None	0.159 ± 0.031	0.131 ± 0.043

Data were expressed as Mean ± SD. R/B indicates red/blue flicker, R/G indicates red/green flicker, and B/G indicates blue/green flicker.

When comparing the effects of light colour between the EMM and MYO groups, multiple comparison test results indicated that there was a significant difference for low, but not for high frequencies. In the MYO group, at 0.20 Hz, VAR were largest in the R/B condition, followed by the R/G condition and then by the B/G condition. At 0.50 Hz, VAR for the R/B and R/G flickers were significantly larger than for the B/G flicker. At 1.00 Hz, the VAR for the R/B flicker were significantly larger than for the R/G and B/G flickers. At stable conditions, the VAR for R/B flicker were significantly larger than for the B/G flicker, but R/G showed no significant difference with both R/B and B/G group. In the EMM group, at 0.20 Hz, VAR for the R/B flicker were significantly larger than for the R/G and B/G flickers. At 0.50 Hz, the VAR for the R/B and R/G flickers were significantly larger than for the B/G flicker.

## Discussion

Artificial light, such as coloured flickering light, in our daily life is quite different from natural light sources and may become light pollution. As previous studies have shown that colour flickers can affect the eye development in animals, we here investigated the potential role of colour flickers in human myopia development and progression. Current study found that both colour and temporal frequency could affect VAR in myopes and emmetropes and that VAR are larger in myopes than in emmetropes. Indeed, higher frequencies were associated with smaller VAR, and R/B colour flickers led to the largest VAR.

### The effect of colour and temporal frequency on VAR

Our study showed that colour and temporal frequency both had significant effects on VAR. VAR were found to decrease significantly as the frequency increased for all colour conditions in both groups, VAR under 5 Hz condition showed no significant difference with VAR under stable light (Fig. 3). At low frequencies (0.20–1.00 Hz in the MYO group and 0.20–0.50 Hz in the EMM group), VAR in the R/B group were larger than the other colour conditions.

When focusing on the target combined with different colours, the focus may not be very accurate because lights of different wavelengths have their focus points at different locations on the retina. However, various text-background colour combinations on electronic visual displays show no significant difference in the accommodative response, which may stem from the fact that different subjects use different focus strategies [23, 24]. Some studies found that light with narrow spectral bandwidth can’t drive accurate accommodation [25, 26]. However, using colour flickers that can provide a dynamic wavelength change, we found that accommodation could occur following a shift to different colours and the colour showed a significant effect on VAR. A bigger wavelength difference leads to a bigger gap in accommodation, thus causing a greater variability in accommodation which may result in more defocus on retina. In the current study, R/B flicker, which had the biggest wavelength difference, led to the biggest accommodative response gap among all colour flickers, and thus the largest VAR in low-frequency conditions. On the contrary, VAR for R/G flicker were larger than for B/G flicker at low-frequency conditions, although the wavelength difference for the R/G flicker was smaller than that for the B/G flicker. This unexpected result may be due to the relative insensitivity of the central fovea of the human eye to blue light and narrow spectral bandwidth of blue light in our study [25, 27]. This implies that the VAR may not simply depend on the wavelength difference, and thus further studies of the relationship between wavelength differences and VAR should be performed in the future.

In the study, VAR decreased as the frequencies increased and showed no significant difference with stable light under 5 Hz light conditions. Previous studies showed light with high flickering frequency (higher than 8 Hz) making less effect on human accommodation [28, 29]. In our study VAR under 5 Hz showed no significant difference with stable light which we regard as control light. However, as the upper limit of detection frequency in our study is 5 Hz, 5 Hz may not be the frequency threshold. Our study found flickering light with low frequency can increase VAR which arouses our interest. It has been shown that colour and temporal frequency of colour flickers can both influence emmetropisation in animals [9, 30–32]. Animal studies revealed that flickering light at high frequencies can cause hyperopia, while myopia can be resulted in at low frequencies [33–35]. The signal at low frequencies may reach deeper levels of the anterior chamber, increasing the secretion and slowing down the outflow of fluid in the eye [36]. Our study showed that VAR at low frequencies are larger than those at high frequencies, and the colour effect is only significant at low frequencies, which is in agreement with the results obtained in animal studies [9, 10]. These animal studies showed that colour played a more important role at low temporal frequencies than for high ones, which is an important clue for emmetropisation [9, 10]. For high frequencies, eyes may not be fast enough to accommodate accurately after colour flickers. Eyes may regard a target with a high temporal flicker frequency as a visual target being in focus [9].

Light emitting diode (LED) lights used most commonly in our life, such as white domestic light is generated by two photons of complementary wavelengths, with a spectrum combined by two peak of waves which is quite different from the natural sun light [37]. The flickering frequencies for common LED lights are 100–120 Hz, which are larger than human critical fusion frequency and seems have rarely effects on human accommodation [38]. However, apart from LED lights with high flickering frequency, color flickering lights with low frequency are also common in our life, such as flickering neon lights, flash lamps and colourful videos. Our study showed the probable side effect of such lights on accommodation and this may be a light pollution for eyes in our life.

### The effect of refractive error on VAR

Our study revealed that VAR were significantly larger in the MYO group than in the EMM group for every colour flicker condition (Fig. 3).

The accommodative response in our study is stimulated by colour flickers accommodative stimulation based on the inherent AMFs. Because of the change in the wavelength from colour flickers, the eyes accommodate quickly to maintain the focus of the current wavelength on the retina, resulting in a fast fluctuation of the lens. Larger VAR in MYO group is in line with previous studies also showing that AMFs for myopic subjects were larger [18, 20]. Increased AMFs can weakly predict myopic progression and may be considered a risk factor for myopia [19]. It has been hypothesized that the retinal defocus resulting from accommodative fluctuation may lead to myopia [39, 40]. Larger intrinsic depth of focus and smaller contrast gradient decrease the sensitivity of blur in myopes, which result in larger accommodative fluctuation for sufficient accommodative response [18, 41, 42]. The defocus on retina accumulate over time and finally accelerate eye axis by mechanism waiting for exploration [39]. Interestingly, studies had found that there was a low correlation between myopia and colour vision deficiency (CVD) [43]. The disability of using color as a clue for accommodation in people with CVD may result in smaller effects of colour flickers on VAR, which lead to lower myopia rate in an unknown mechanism [44].

Colour flickers are ubiquitous in our life, such as the colourful neon lights on the streets, colourful videos on video display terminals. Our study showed that low temporal frequency and R/B colour flicker result in large VAR. So, watching colourful videos and using colourful flicking neon lights in daily life may accelerate myopia progression in young adults. The effect of colour flickers on VAR draw our attention and focus to these flickering lights in our normal, everyday life.

To the best of our knowledge, the present study is the first to test the hypotheses from animal studies in humans, that colour flickers can influence myopia development and progression in myopes and emmetropes. One limitation of our study was that the largest detection frequency of the equipment we used to record the accommodation response was only 5 Hz, which may not be large enough to identify the frequency threshold that affects VAR. Thus, further studies are needed to confirm the functional frequency threshold and find the safety frequencies of colour flickers. In addition, since this study was performed only on adults with normal colour vision, further research could detect the influence of colour flickers on myopia development in children and people with CVD.

## Conclusions

This study investigated the VAR on myopes and emmetropes in response to colour flicker at different temporal frequencies. Lower frequencies led to larger VAR. Colour flicker was shown to only have an effect at low frequencies, and VAR for R/B colour flicker were the largest. The larger VAR found in the myopes and the effects of colour flicker and temporal frequency on VAR necessitate further study into the effects of colour flickers on myopia development and progression.

## Data Availability

The data are available from the corresponding author upon reasonable request.

## Abbreviations

VAR: Variability of the accommodation response
LCA: Longitudinal chromatic aberration
AMFs: Accommodative microfluctuations
EMM: Emmetropes
MYO: Myopes
D: Diopters
SE: Spherical equivalent
LSD: Least significance difference
SPSS: Statistical product and service solutions
LED: Light emitting diode
CVD: Colour vision deficiency

## References

[1] Dolgin E (2015). The myopia boom. Nature..

[2] Holden BA, Fricke TR, Wilson DA (2016). Jong M, Naidoo KS, el al. Global prevalence of myopia and high myopia and temporal trends from 2000 through 2050. Ophthalmology..

[3] Morgan IG, French AN, Ashby RS, Guo X, Ding X (2018). The epidemics of myopia: Aetiology and prevention. Prog Retin Eye Res.

[4] Lingham G, Mackey DA, Lucas R, Yazar S (2020). How does spending time outdoors protect against myopia? A review. Brit J Ophthalmol.

[5] Morgan IG, Rose KA (2019). Myopia: is the nature-nurture debate finally over?. Clin Exp Optom..

[6] Guggenheim JA. Northstone K, McMahon G, Ness AR, Deere K, el al. Time outdoors and physical activity as predictors of incident myopia in childhood: a prospective cohort study. Invest Ophth Vis Sci. 2012;53:2856–65. 10.1167/iovs.11-9091 PMC3367471 22491403

[7] Wu PC, Chen CT, Lin KK, Sun CC, Kuo CN (2018). Myopia prevention and outdoor light intensity in a school-based cluster randomized trial. Ophthalmology.

[8] Gawne TJ, Siegwart JT, Ward AH, Norton TT (2017). The wavelength composition and temporal modulation of ambient lighting strongly affect refractive development in young tree shrews. Exp Eye Res.

[9] Rucker F, Britton S, Taylor C (2018). Color and temporal frequency sensitive eye growth in Chick. Invest Ophth Vis Sci..

[10] Tian T, Zou L, Wu S, Liu H, Liu R (2019). Wavelength defocus and temporal sensitivity affect refractive development in Guinea pigs. Invest Ophth Vis Sci..

[11] Marcos S, Burns SA, Moreno-Barriusop E, Navarro R (1999). A new approach to the study of ocular chromatic aberrations. Vis Res.

[12] Charman WN, Tucker J (1978). Accommodation and color. J Opt Soc Am.

[13] Campbell FW, Robson JG, Westheimer G (1959). Fluctuations of accommodation under steady viewing conditions. J Physiol.

[14] Charman WN, Heron G (1988). Fluctuations in accommodation: a review. Ophthal Physiol Opt..

[15] Lin H, Drobe B, Jin W, Lin M, Chen Y (2016). el al. Effects of near addition lenses and prisms on accommodative microfluctuations in Chinese children. Optometry Vision Sci.

[16] Sreenivasan V, Irving EL, Bobier WR (2011). Effect of near adds on the variability of accommodative response in myopic children. Ophthal Physiol Opt..

[17] Xu J, Lu X, Zheng Z, Bao J, Singh N, et al. The effects of spatial frequency on the accommodative responses of myopic and emmetropic Chinese children. Transl Vis Sci Techn. 2019;8:65. 10.1167/tvst.8.3.65 PMC6602141 31293819

[18] Day M, Gray LS, Seidel D, Strang NC (2009). The relationship between object spatial profile and accommodation microfluctuations in emmetropes and myopes. J Vision.

[19] Langaas T, Riddell PM (2012). Accommodative instability: relationship to progression of early onset myopia. Clin Exp Optom.

[20] Langaas T, Riddell PM, Svarverud E, Ystenaes AE, Langeggen I (2008). el al. Variability of the accommodation response in early onset myopia. Optometry Vision Sci..

[21] Day M, Strang NC, Seidel D, Gray LS (2008). Effect of contact lenses on measurement of the accommodation microfluctuations. Ophthal Physiol Opt..

[22] Sheppard AL, Davies LN (2010). Clinical evaluation of the grand Seiko auto ref/Keratometer WAM-5500. Ophthal Physiol Opt..

[23] Atchison DA, Strang NC, Stark LR (2004). Dynamic accommodation responses to stationary colored targets. Optometry Vision Sci..

[24] Jiménez R, Redondo B, Molina R, Martínez-Domingo MÁ, Hernández-Andrés J (2020). el al. Short-term effects of text-background color combinations on the dynamics of the accommodative response. Vis Res.

[25] Aggarwala KR, Mathews S, Kruger ES, Kruger PB (1995). Spectral bandwidth and ocular accommodation. J Opt Soc Am A Opt Image Sci Vis.

[26] Aggarwala KR, Nowbotsing S, Kruger PB (1995). Accommodation to monochromatic and white-light targets. Invest Ophth Vis Sci..

[27] Wald G (1967). Blue-blindness in the normal fovea. J Opt Soc Am.

[28] Chauhan K, Charman WN, Halnan AM, Kelly CM, Loughlin A (1992). Time-averaged accommodation response to flickering stimuli. Ophthal Physiol Opt.

[29] Flitcroft DI (1991). Accommodation and flicker: evidence of a role for temporal cues in accommodation control?. Ophthal Physiol Opt.

[30] Rucker F, Henriksen M, Yanase T, Taylor C (2018). The role of temporal contrast and blue light in emmetropization. Vis Res.

[31] Rucker F, Britton S, Spatcher M, Hanowsky S (2015). Blue light protects against temporal frequency sensitive refractive changes. Invest Ophth Vis Sci..

[32] Rucker FJ, Wallman J (2012). Chicks use changes in luminance and chromatic contrast as indicators of the sign of defocus. J Vision..

[33] Di Y, Liu R, Chu RY, Zhou XT, Zhou XD (2013). Myopia induced by flickering light in Guinea pigs: a detailed assessment on susceptibility of different frequencies. Int J Ophthalmol.

[34] Li B, Luo X, Li T, Zheng C, Ji S (2016). el al. Effects of constant flickering light on refractive status, 5-HT and 5-HT2A receptor in Guinea pigs. PLoS One.

[35] Zhi Z, Pan M, Xie R, Xiong S, Zhou X (2013). The effect of temporal and spatial stimuli on the refractive status of Guinea pigs following natural emmetropization. Invest Ophth Vis Sci..

[36] Crewther SG, Barutchu A, Murphy MJ, Crewther DP (2006). Low frequency temporal modulation of light promotes a myopic shift in refractive compensation to all spectacle lenses. Expl Eye Res.

[37] Behar-Cohen F, Martinsons C, Viénot F, Zissis G, Barlier-Salsi A (2011). Light-emitting diodes (LED) for domestic lighting: any risks for the eye?. Prog Retin Eye Res.

[38] Lehman B, Wilkins A, Berman S, Poplawski M, Miller NJ (2011). Proposing measures of flicker in the low frequencies for lighting applications. Leukos..

[39] Wallman J, Winawer J (2004). Homeostasis of eye growth and the question of myopia. Neuron..

[40] Wang D, Chun RK, Liu M, Lee RP, Sun Y (2016). Optical defocus rapidly changes choroidal thickness in schoolchildren. PLoS One.

[41] Day M, Seidel D, Gray LS, Strang NC (2009). The effect of modulating ocular depth of focus upon accommodation microfluctuations in myopic and emmetropic subjects. Vis Res.

[42] Day M, Strang NC, Seidel D, Gray LS, Mallen EA (2006). Refractive group differences in accommodation microfluctuations with changing accommodation stimulus. Ophthal Physiol Opt..

[43] Qian YS, Chu RY, He JC, Sun XH, Zhou XT (2009). el al. Incidence of myopia in high school students with and without red-green color vision deficiency. Invest Ophthalmol Vis Sci.

[44] Fincham EF (1953). Defects of the colour-sense mechanism as indicated by the accommodation reflex. J Physiol.

